# Analysis of mammograms using artificial intelligence to predict response to neoadjuvant chemotherapy in breast cancer patients: proof of concept

**DOI:** 10.1007/s00330-021-08306-w

**Published:** 2021-10-15

**Authors:** I. Skarping, M. Larsson, D. Förnvik

**Affiliations:** 1 Division of Oncology, Department of Clinical Sciences, Lund University, Lund, Sweden; 2 Department of Clinical Physiology and Nuclear Medicine, Skane University Hospital, Lund, Sweden; 3 Eigenvision AB, Malmö, Sweden; 4 Medical Radiation Physics, Department of Translational Medicine, Lund University, Skane University Hospital, Malmö, Sweden

**Keywords:** Breast neoplasms, Diagnostic imaging, Neoadjuvant therapy, Artificial intelligence

## Abstract

**Objectives:**

In this proof of concept study, a deep learning–based method for automatic analysis of digital mammograms (DM) as a tool to aid in assessment of neoadjuvant chemotherapy (NACT) treatment response in breast cancer (BC) was examined.

**Methods:**

Baseline DM from 453 patients receiving NACT between 2005 and 2019 were included in the study cohort. A deep learning system, using the aforementioned baseline DM, was developed to predict pathological complete response (pCR) in the surgical specimen after completion of NACT. Two image patches, one extracted around the detected tumour and the other from the corresponding position in the reference image, were fed into a classification network. For training and validation, 1485 images obtained from 400 patients were used, and the model was ultimately applied to a test set consisting of 53 patients.

**Results:**

A total of 95 patients (21%) achieved pCR. The median patient age was 52.5 years (interquartile range 43.7–62.1), and 255 (56%) were premenopausal. The artificial intelligence (AI) model predicted the pCR as represented by the area under the curve of 0.71 (95% confidence interval 0.53–0.90; *p* = 0.035). The sensitivity was 46% at a fixed specificity of 90%.

**Conclusions:**

Our study describes an AI platform using baseline DM to predict BC patients’ responses to NACT. The initial AI performance indicated the potential to aid in clinical decision-making. In order to continue exploring the clinical utility of AI in predicting responses to NACT for BC, further research, including refining the methodology and a larger sample size, is warranted.

**Key Points:**

*• We aimed to answer the following question: Prior to initiation of neoadjuvant chemotherapy, can artificial intelligence (AI) applied to digital mammograms (DM) predict breast tumour response?*

*• DMs contain information that AI can make use of for predicting pathological complete (pCR) response after neoadjuvant chemotherapy for breast cancer.*

*• By developing an AI system designed to focus on relevant parts of the DM, fully automatic pCR prediction can be done well enough to potentially aid in clinical decision-making.*

## Introduction

Neoadjuvant chemotherapy (NACT) for breast cancer (BC) is increasingly used for patients with early-stage disease who are eligible for chemotherapy [1]. Advantages include assessment of treatment response with the option to continuously alter the systemic regime and provide an individualised prognosis post-NACT. From a surgical perspective, NACT enables less invasive surgery, both in terms of causing tumour shrinkage that would permit breast-conserving surgery and lowering the rates of axillary dissections due to recent treatment changes with the use of post-NACT sentinel lymph node biopsies [2, 3]. In addition, the recently introduced concept of salvage adjuvant chemotherapy for patients who do not achieve pathological complete response (pCR) further utilises the NACT setting [4–6].

In a clinical routine, assessment of disease stage (T- and N-stage) for BC patients undergoing NACT is based on imaging of the breast and the axilla. Common imaging includes digital mammograms (DM) and axillary ultrasound (US), whereas breast US, magnetic resonance imaging (MRI) and/or positron emission tomography/computed tomography (PET/CT) are less often used [7]. Imaging can predict a high degree of disease progression but has discouraging results when predicting pCR, a surrogate for survival [8, 9]. In clinical practice and research, different approaches can be used to monitor tumour responses during NACT: (1) clinical exam (most commonly used), (2) dynamic changes in tumour size as measured by imaging, (3) sequential biopsy of the tumour to evaluate change in biomarkers (often proliferation marker Ki67) and (4) molecular biomarkers in blood samples drawn over the time course of treatment [10, 11].

Previously, we investigated the impact of breast density on treatment response prediction as a means to extract additional data from the clinical DM but did not obtain any conclusive results by using this method [12, 13]. Going beyond a mere value of breast density, evidence that certain breast parenchymal patterns and tumour appearances, which could potentially have an impact on treatment response, are associated with the breast tissue milieu have been demonstrated [14, 15].

In this proof of concept study [16], we introduce a deep learning–based method for automatic DM analysis as a tool to aid in treatment response assessment. Convolutional neural networks (CNNs) have shown outstanding performance in image recognition tasks as this process automatically learns feature representation in a general manner from pixels in medical images according to corresponding class annotations [17]. Our hypothesis is that treatment response is affected by breast parenchymal patterns and tumour appearances as reflected by different grey-level pixel presentations or the features of images that can be deciphered using a CNN. The aim of this work was to develop a DM-based CNN model that will provide the discriminative power of pCR. The clinical question at hand can be asked: ‘Prior to initiation of neoadjuvant chemotherapy, can we predict tumour responses utilising artificial intelligence (AI) in DM?’.

## Materials and methods

### Cohort

**Fig. 1:**
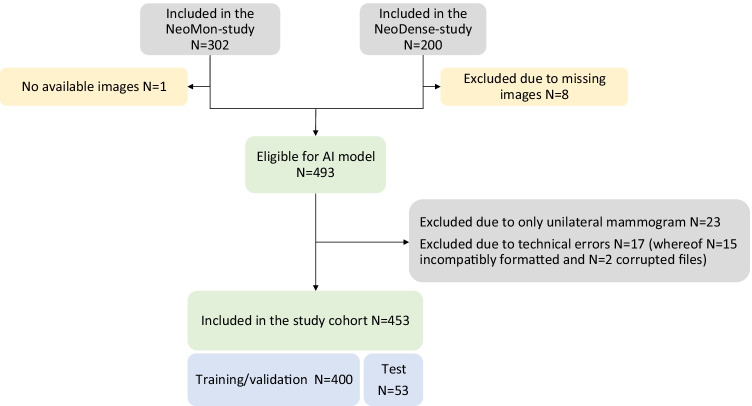
Patient flow chart

The cohort consisted of female BC patients undergoing NACT (chemotherapy, and in cases of human epidermal growth factor receptor 2 [HER2] positivity, combined with HER2 blockade) for BC in Sweden from 2005 to 2019. A total of 493 patients were eligible for the AI model. The reasons for exclusion (50 patients) are pictured in Fig. 1. The study cohort (453 patients) consisted of a retrospective (*N* = 258, treatment period 2005–2016) and a prospective cohort (*N* = 195, treatment period 2014–2019) as previously described [18]. The inclusion criteria for both cohorts were female patients treated with NACT undergoing the intended breast surgery. Medical charts and study-specific patient questionnaires (filled out upon diagnosis) were reviewed and data on patients’ characteristics were retrieved.

In total, 90% (*N* = 408) of the patients received a chemotherapy regimen consisting of a combination of epirubicin and cyclophosphamide (EC)/fluorouracil, epirubicin and cyclophosphamide (FEC) and docetaxel/paclitaxel. Additionally, 8% (*N* = 38) of the patients received taxane-only NACT regimen and a total of 1% (*N* = 4) of the patients received FEC/EC only. The remainder 1% (*N* = 3) received various other regimens. For the patients with HER2 positive tumours (*N* = 134), *N* = 89 (66%) received trastuzumab, *N* = 43 (32%) received trastuzumab and pertuzumab, and the remaining two (1%) received no HER2 targeted treatment.

Information about tumour pathology from the surgical specimen following NACT was derived from clinical-pathological reports. In accordance with national guidelines, tumour hormone receptor positivity was defined according to staining positive in > 10% of the tumour cells with immunohistochemistry (IHC), and HER2 status was defined as either 3 + with IHC and/or amplified with fluorescence in situ hybridisation [11]. The proliferation marker Ki67 (reported as a percentage from 0 to 100%), was considered highly proliferative when Ki67 > 20%, and otherwise low proliferative [11]. The following definition for pCR was used: the absence of any residual invasive cancer in the resected breast after surgery as well as all sampled regional lymph nodes following completion of NACT [19].

All women with any baseline DMs were included in this study. From the eligible patients (*N* = 493), drawn randomly while maintaining the cohort pCR ratio, a test set of 60 (12%) patients were set aside for final assessment. The development and execution of the AI model led to the exclusion of 40 (8%) patients; 23 (5%) were excluded because only one breast had been imaged and 17 (3%) were excluded due to technical errors of which 15 (3%) were formatted incorrectly and two (0.4%) had corrupt files (Fig. 1). Four-hundred (88%) patients were used for model training and validation in an 80/20 ratio, and 53 (12%) for final assessment (of the original *N* = 60 in the reserved test set) (Table 1). This study was designed in accordance with the Strengthening of the Reporting of Observational Studies (STROBE) guidelines [20].

**Table 1:** Patient and tumour characteristics at baseline (whole cohort and stratified according to pathological complete response [pCR] status)

		All	pCR	Non-pCR
Total, *N*		453	95 (21.0%)	358 (79.0%)
Age (years) median (IQR)		52.5 (43.7–62.1)	53.1 (43.9–62.6)	52.4 (43.7–62.1)
BMI (kg/m^2^), median (IQR)		25 (23–29)	25 (23–29)	25 (23–29)
Menopausal status	Premenopausal	255 (56.3%)	52 (54.7%)	203 (56.7%)
	Postmenopausal	198 (43.7%)	43 (45.3%)	155 (43.3%)
Any births	No children	62 (13.7%)	10 (10.5%)	52 (14.5%)
	1 or more children	390 (86.1%)	85 (89.5%)	305 (85.2%)
	Missing	1 (0.2%)	0 (0.0%)	1 (0.3%)
Ever hormone replacement therapy	Yes	57 (12.6%)	14 (14.7%)	43 (12.0%)
	No	393 (86.8%)	81 (85.3%)	312 (87.2%)
	Missing	3 (0.7%)	0 (0.0%)	3 (0.8%)
Mammographic density, BI-RADS	A	22 (4.9%)	7 (7.4%)	15 (4.2%)
	B	174 (39.1%)	40 (42.1%)	137 (38.3%)
	C	205 (45.3%)	39 (41.1%)	166 (46.4%)
	D	49 (10.8%)	9 (9.5%)	40 (11.2%)
Tumour size (mm), median (IQR)		30.0 (22.0–40.0)	26.5 (20.0–34.5)	31.5 (22.5–40.0)
Oestrogen receptor	Positive (> 10%)	274 (60.5%)	24 (25.3%)	250 (69.8%)
	Negative (≤ 10%)	175 (38.6%)	70 (73.7%)	105 (29.3%)
	Missing	4 (0.9%)	1 (1.1%)	3 (0.8%)
Progesterone receptor	Positive (> 10%)	224 (49.4%)	15 (15.8%)	209 (58.4%)
	Negative (≤ 10%)	224 (49.4%)	79 (83.2%)	145 (40.5%)
	Missing	5 (1.1%)	1 (1.1%)	4 (1.1%)
HER2 receptor	Positive	134 (29.6%)	51 (53.7%)	83 (23.2%)
	Negative	310 (68.4%)	42 (44.2%)	268 (74.9%)
	Missing	9 (2.0%)	2 (2.1%)	7 (2.0%)
Proliferation (Ki67)	High (> 20%)	361 (79.7%)	82 (86.3%)	279 (77.9%)
	Low (≤ 20%)	51 (11.3%)	3 (3.2%)	48 (13.4%)
	Missing	41 (9.1%)	10 (10.5%)	31 (8.7%)
St. Gallen subtype	Luminal A–like	45 (9.9%)	0 (0.0%)	45 (12.6%)
	Luminal B–like	136 (30.0%)	7 (7.4%)	129 (36.0%)
	HER2-positive	134 (29.6%)	51 (53.7%)	83 (23.2%)
	Triple-negative	113 (24.9%)	34 (35.8%)	79 (22.1%)
	Missing	25 (5.5%)	3 (3.2%)	22 (6.1%)
Node status	Positive	307 (67.8%)	63 (66.3%)	244 (68.2%)
	Negative	76 (16.8%)	23 (24.2%)	53 (14.8%)
	Missing	70 (15.5%)	9 (9.5%)	61 (17.0%)

*pCR*, pathological complete response; *BMI*, body mass index; *HER2*, human epidermal growth factor receptor 2; *IQR*, interquartile range

All procedures performed in studies involving human participants were in accordance with the ethical standards of the institutional and/or national research committee, and with the 1964 Helsinki declaration and its later amendments or comparable ethical standards. The study was approved by the Regional Ethics Committee in Lund, Sweden (committee’s reference number: 2014/13, 2014/521 and 2016/521).

### Imaging

DM were retrieved from local picture archiving system (Sectra AB) and/or prospectively stored on a local server. The 2514 processed DM (training *N* = 1796 DM, validation *N* = 422 DM and test set *N* = 296 DM) selected for analysis originated from the following vendors: (1) GE Healthcare (31%), (2) Philips Healthcare (16%) and (3) Siemens Healthineers (52%). All patients had sets of either six or four images with an average of 5.5 images acquired from both breasts and all available views (cranio-caudal and medio-lateral oblique for all patients and additional lateral-medial views in the set of six images). The important image parameters are collected from the standardised meta-information, i.e. Digital Imaging and Communications in Medicine (DICOM) tags. When this was not standardised, we encountered technical issues as in *N* = 15 cases. All operations are invariant to any remaining differences.

### Neural networks

**Fig. 2:**
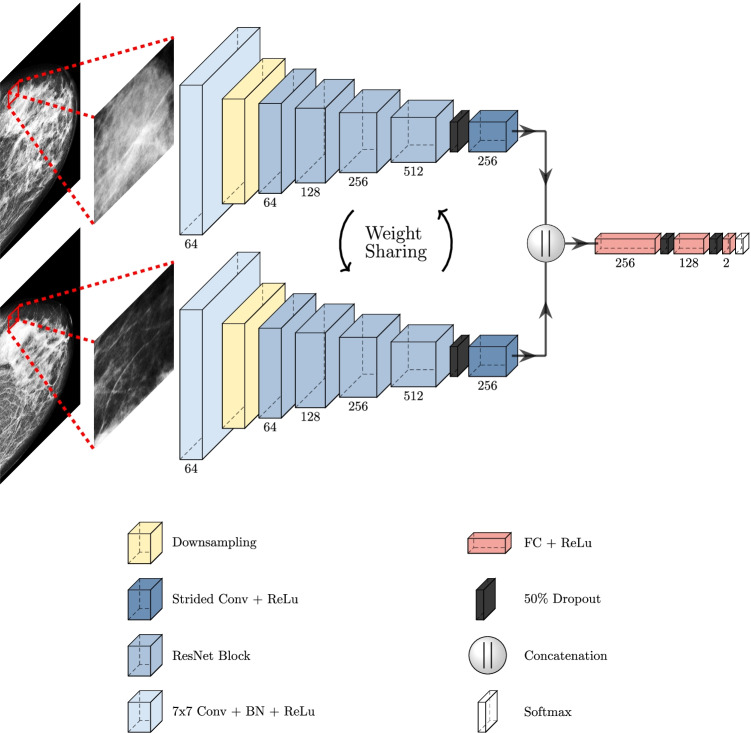
Classification model architecture. Image patches from the detected tumour and corresponding position in the reference image were processed in two parallel pathways. The feature output from the two pathways was concatenated and processed by a series of fully connected layers and a final soft-max layer. The numbers shown in the image denotes the size of the feature dimension

The deep learning system used to predict pCR in DM consists of two main steps: (1) a network for detection tumours is first applied to the DM and (2) image patches are extracted around the detected tumour in addition to the same position in the reference image (contralateral cancer-free breast). The two image patches are fed into a classification network (Fig. 2) that predicted pCR. By extracting smaller image patches of interest, the classification network is forced to make predictions based on what we hypothesised to be relevant information instead of overfitting the information to irrelevant input.

For detection, a detection transformer (DETR) model was selected [21]. The model uses the whole DM as input and predicts the location and size of tumours in the form of bounding boxes. Apart from changing the number of output classes to one tumour, we followed the same training and inference procedures as described in the original paper [21]. For training, we used the publicly available Curated Breast Imaging Subset of DDSM (CBIS-DDSM) consisting of scanned film mammography studies paired with bounding boxes of tumours [22]. In total, we used 1485 images during training and validation of the detection network. Before being input to the model, histogram equalisation was performed on the images. This step was crucial for allowing the detection model to perform well on the images in the cohort presented in this paper. The equalisation was done to increase the contrast in the images as well as decrease differences between images from different vendors. This is done by using the normalised histogram of the original image intensities to remap the pixel intensities [23]. The resulting image will have a uniform distribution of image intensities. Equalisation was done on the range of intensities available in the original image, excluding zero.

The classification network consists of two parallel pathways in which the first one processes the image patch extracted from the detected tumour, and the second one processes the patch extracted from the reference image. The two pathways have the same base structures as ResNet18 [24] with a strided convolution at the end, which reduces the spatial size of the feature maps to 1 × 1. The features from the two pathways are then concatenated and processed through a series of fully connected and dropout layers to finally output classification scores for pCR, and non-pCR, a visualisation of the network structure can be seen in Fig. 2.

The classification network processed the DM in the original resolution of 0.085 and 0.085 mm per pixel for Siemens and Philips systems and 0.1 and 0.1 mm per pixel for GE systems. The size of the image patches input was 224 × 224 pixels. For the images in which multiple tumours were detected, a random one was chosen during training, whereas for images without detected tumours, a random area was selected in the foreground of the imaging areas within the breast contour. The training was done using cross-entropy loss and stochastic gradient descent with a learning rate 1e^−3^, momentum 0.9, weight decay 1e^−4^ and dropout rate of 0.5 for all dropout layers. Training was done for 300 epochs, and the weights from the epoch with lowest validation loss were used. During inference, the classification network was applied to all detected tumours averaging the output. For the final estimated patient pCR probability, the output for all available views was averaged.

### Statistical analyses

We summarised cohort baseline characteristics for the whole cohort as absolute values and as percentual shares that were split by pCR status with pCR binary outcome. Receiver operating characteristics (ROC) curves were constructed, and a two-sided Mann–Whitney *U* test was used for statistical significance testing. Statistical significance was defined as *p* < 0.05.

For both descriptive and analytic statistics, IBM SPSS Statistics for Windows, version 26 (IBM Corp.) was used.

## Results

### Descriptive results

The patient and tumour characteristics of the 453 patients included in this study are presented in total and according to pCR in Table 1. A total of 95 patients (21%) achieved pCR. The median age was 52.5 years (interquartile range [IQR] 43.7–62.1), and 255 (56%) patients were premenopausal. The median tumour size at baseline was 30.0 mm (IQR 22.0–40.0); the patients in the pCR group presented somewhat smaller tumours (26.5 mm, IQR 20.0–34.5) in comparison to the non-pCR group (31.5 mm, IQR 22.5–40.0). The majority of the patients had highly proliferative tumours (*N* = 361, 80%) and presented with nodal metastases at diagnosis (*N* = 307, 68%). In terms of the St. Gallen subtype, patients with luminal A–like tumours were in the minority (as is to be expected given the criteria for receiving NACT), and none of these patients achieved pCR. Correspondingly, descriptive statistics for the randomly drawn test set (*N* = 53) is presented in Table 2; the test set shows similar patients and tumour characteristics of the cohort as a whole.

**Table 2:** Patient and tumour characteristics at baseline (test set, *N* = 53)

		*N* (%)
pCR	Yes	11 (20.8%)
	No	42 (79.2%)
Age (years) median (IQR)		55.5 (45.8–65.1)
BMI (kg/m^2^), median (IQR)		26 (24–29)
Menopausal status	Premenopausal	25 (47.2%)
	Postmenopausal	28 (52.8%)
Any births	No children	4 (7.5%)
	1 or more children	49 (92.5%)
	Missing	0 (0.0%)
Ever hormone replacement therapy	Yes	6 (11.3%)
	No	47 (88.7%)
	Missing	0 (0.0%)
Mammographic density, BI-RADS	A	2 (3.8%)
	B	26 (49.1%)
	C	19 (35.8%)
	D	6 (11.3%)
Tumour size (mm), median (IQR)		30.0 (22.0–38.0)
Oestrogen receptor	Positive (> 10%)	35 (66.0%)
	Negative (≤ 10%)	17 (32.1%)
	Missing	1 (1.9%)
Progesterone receptor	Positive (> 10%)	29 (54.7%)
	Negative (≤ 10%)	22 (41.5%)
	Missing	2 (3.8%)
HER2 receptor	Positive	16 (30.2%)
	Negative	36 (67.9%)
	Missing	1 (1.9%)
Proliferation (Ki67)	High (> 20%)	42 (79.2%)
	Low (≤ 20%)	8 (15.1%)
	Missing	3 (5.7%)
St. Gallen subtype	Luminal A–like	7 (13.2%)
	Luminal B–like	16 (30.2%)
	HER2-positive	16 (30.2%)
	Triple-negative	13 (24.5%)
	Missing	1 (1.9%)
Node status	Positive	40 (75.5%)
	Negative	5 (9.4%)
	Missing	8 (15.1%)

*pCR*, pathological complete response; *BMI*, body mass index; *HER*, human epidermal growth factor receptor; *IQR*, interquartile range

### AI

**Fig. 3:**
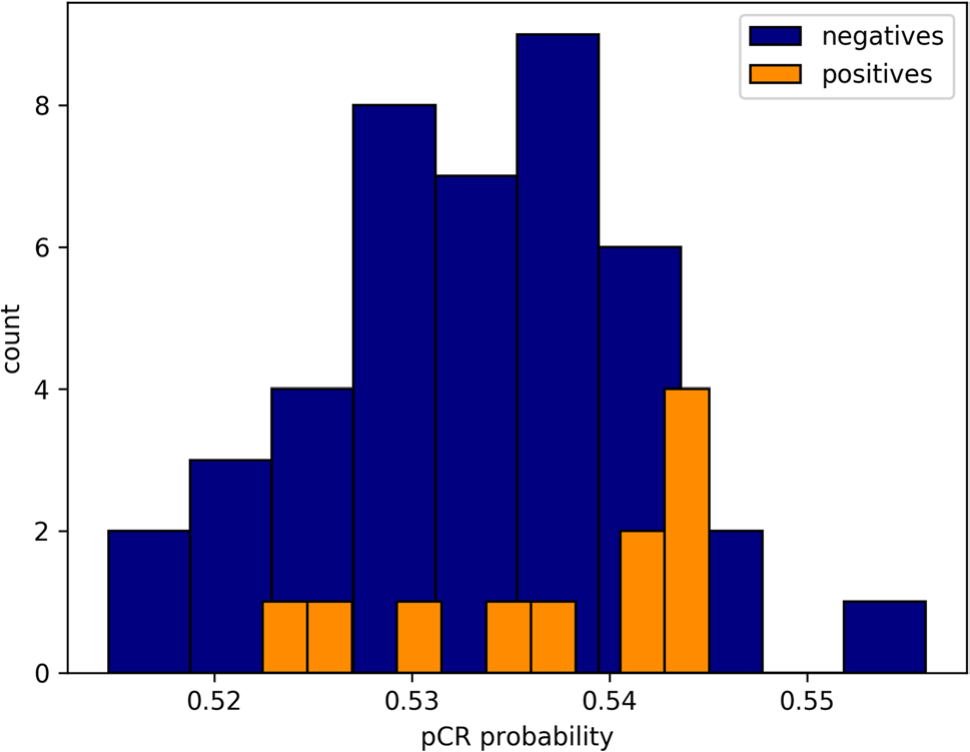
Distribution of the pathological complete response (pCR) probability scores in the test set

**Fig. 4:**
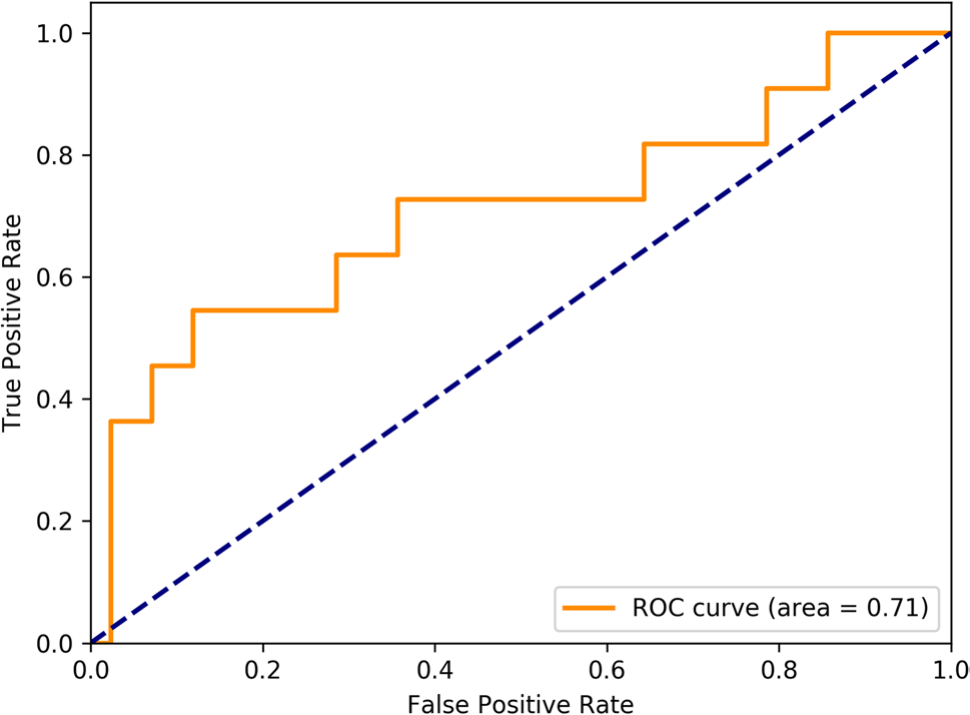
Receiver operating characteristics (ROC) curve for the artificial intelligence (AI) model

The AI output probability pCR scores for the test set are shown in Fig. 3, and the associated ROC curve is illustrated in Fig. 4. The AI model showed a performance of predicting pCR as represented by the area under the curve [AUC] of 0.71 (95% confidence interval [CI], 0.53–0.90; *p* = 0.036). The sensitivity at a fixed false-positive rate of 0.10 (90% specificity) was 0.46. Please note that false-positive here refers to AI predicting pCR where in fact the patient did not accomplish pCR.

## Discussion

Prediction of treatment response, upfront or as early as possible, is important in order to offer BC patients individualised treatment. Currently, evaluation of responses to NACT includes anatomical imaging, functional imaging (metabolic evaluation through PET/CT), and possibly biomarker evaluation [10, 25]. To the best of the authors’ knowledge, this study is the first one investigating the use of AI on baseline DM to predict treatment responses. In this report, we present results of a deep learning–based method on baseline DM and its capability to subsequently identify patients who achieved pCR, resulting in an AUC of 0.71.

### AI and treatment response evaluation

Tahmassebi et al. conducted a study using machine learning based on both pre- and during-NACT MRI (*N* = 38) with residual cancer burden as an outcome measure (with class zero being defined as pCR), which yielded an AUC of 0.86 [26]. A study by Qu et al. presented results of a deep learning–based method applied to MRI (*N*/training = 244, *N*/validation = 58) using pCR as an outcome measure and showed an AUC of 0.55 using pre-NACT data in comparison to an AUC of 0.97 when using post-NACT data or the combination of both pre- and post-NACT MRI [27]. Sutton et al. applied machine learning to pre- and post-NACT MRI (*N*/training = 222, *N*/validation = 56) and showed an AUC between 0.78 and 0.83. In the latter model, the molecular subtype was added to radiomics [28]. From the I-SPY TRIAL breast MRI database, an implemented CNN algorithm on MRI (*N* = 131) showed an AUC of 0.72 [29]. Similarly, CNN used in a pre-NACT MRI study by Ha et al. (*N* = 141) showed an AUC as high as 0.98 [30]. Cain et al. built multivariate machine learning models (logistic regression and a support vector machine) based on pre-NACT MRI (*N*/training = 144, *N*/ validation = 144), which resulted in an AUC of 0.71 [31]. Nevertheless, our results suggest that our AI model on DM is in the range of those based on pre-NACT MRI. The most obvious advantages of DM are easy accessibility worldwide in contrast to the expensive and more complicated imaging methods of MRI and PET/CT.

### Predictive factors for NACT response

It is well known that different BC subtypes with their heterogeneous biology respond differently to NACT [32]. Generally, the most aggressive BC subtypes are associated with higher pCR rates [32]. On the other hand, the relevance of pCR as an outcome measure is less certain for luminal BC, which is often considered a less aggressive subtype [32, 33]. In addition to BC subtype and immunohistochemical parameters, immunological markers (such as tumour-infiltrating lymphocytes), tumour-genetic profiles (which are commonly used in the adjuvant setting), and immune-associated signatures hold predictive information at baseline [34–36]. However, the tumour and its relation to the surrounding tissue must be taken into account. The local microenvironment and the systemic host characteristics also influence tumour response, and these properties are not routinely included in medical decision-making and treatment algorithms [11, 37]. Increased mammographic density, MRI background parenchymal enhancement, higher age and higher body mass index have been suggested to be associated with lower rates of pCR [13, 38–40]. In addition, multiple studies have investigated dynamic predictive factors, for example, predicting pCR status by considering a change in various biomarkers, such as tumour immune microenvironments [41], measurements of cell loss [42] and circulating tumour cells [43]. Many tumour response studies using structural and functional imaging studies are published using both conventional and state-of-the-art imaging, including mammography, tomosynthesis, ultrasound, MRI, PET and shear wave elastography [44–47]. Evaluating AI in breast tomosynthesis would be an interesting line of research for future studies. In order to fine-tune predictive information, many nomograms have been developed that consider multiple parameters aiming to optimise precision in estimation of response to NACT [48]. Recently, the concept has been further developed by evaluating the predictive performances of machine learning using clinical and pathological data [49].

### Implications of identifying pCR/non-pCR

In order to individualise NACT treatment, more biomarkers, including imaging biomarkers, are needed. Tools to early identify responders from non-responders could aid clinical decision-making, motivate patients to continue treatment, and enable the concept of response-guided treatment as introduced in the GeparTrio study [50]. Since response-guided treatment is currently lacking convincing evidence of its benefits, the common strategy is to complete NACT unless evident progression or intolerable side effects occur [11, 37]. Early identification of patients who are not likely to achieve pCR after subsequently administrated NACT has the potential to improve tailored treatment and escalate/de-escalate treatment accordingly. On the other hand, in the post-NACT setting, the potential clinical gain is mostly a surgical matter; if imaging in combination with minimally invasive procedures could lead to a considerably high degree of correctly identified pCR, further invasive surgery for these patients may not be needed.

### Digital mammograms: AI versus breast radiologists

To evaluate the performance of our AI model in relation to the performance of radiologists, seven experienced radiologists jointly reached an AUC of 0.71 in correctly discriminating between pCR and non-pCR (unpublished data from the NeoDense trial [12]) for the post-NACT time point by evaluating DM. The baseline NeoDense study-specific protocol was not designed for the radiologists to estimate subsequent pCR status after completion of NACT; therefore, a more direct comparison between performances at the pre-NACT time point was not possible. Also, comparison with MRI is not possible since this modality is not currently used for this purpose at the study sites.

### Strengths and limitations

We present the results of a relatively large BC patient cohort who received NACT according to clinical routine. Conventional imaging was used according to the local routine at the time being; thus, MRI, as used by many other researchers, was not available. While hindering direct comparison, the use of DM makes our study unique since, to the best of our knowledge, no literature is available concerning AI application to baseline DM to predict treatment response during NACT for BC patients. Before AI training, a test set of patients were set aside for final assessment, enhancing validity of our results. The concerns with a binary output as pCR must be briefly acknowledged. Many post-NACT pathological assessment scores also reflect partial responses, possibly providing a more nuanced prognosis. The importance of these results is most evident when considering salvage adjuvant chemotherapy for which residual cancer burden score 1 (“near-pCR”) shows as good an outcome as patients who achieved a pCR [51]. Nevertheless, convincingly, pCR is still the most widely accepted endpoint in NACT studies.

A limitation of our study is the heterogeneous cohort in terms of both BC subtype and time period for NACT treatment. Unfortunately, the cohort was not large enough to perform subgroup analyses according to BC subtype since AI modelling demands a large number of images. Here, we shortly address possible concerns of the long recording period (2005–2019) and possible changes in NACT treatment during this time period. For both cohorts, the standard NACT contained series of FEC or EC followed by series of taxanes (docetaxel or paclitaxel) and, in the case of HER2-positive tumour, combined with HER2 blockade (trastuzumab/pertuzumab). Thus, the NACT regimen was consistent during the recording time and we therefore believe this to be of minor impact.

### Future aspects

Next, we will train AI using dynamic DM from three time points during NACT and further explore explainable AI by identifying the areas on the mammograms that AI find most informative to generate “heat maps.” In addition, the concept of AI-guided response evaluation during NACT can be applied to other medical images for other organs.

## Conclusion

In conclusion, our study describes an AI platform using baseline DM to predict the response to NACT in BC patients. The initial AI performance presents the potential to aid in the clinical decision-making. In order to continue exploring the clinical utility of AI in predicting response to NACT for BC, further research including refined methodology and a larger sample size is warranted. Overall, our proof-of-concept study of the response evaluation highlights an important area of AI research in BC. In addition, our study might prompt future studies in NACT-treated cancer patients in general and, therefore, has implications beyond BC.

## Abbreviations

AI: Artificial intelligence
AUC: Area under the curve
BC: Breast cancer
CI: Confidence interval
CNN: Convolutional neural network
DM: Digital mammograms
EC: Epirubicin and cyclophosphamide
FEC: Fluorouracil, epirubicin and cyclophosphamide
HER2: Human epidermal growth factor receptor 2
IHC: Immunohistochemistry
IQR: Interquartile range
MRI: Magnetic resonance imaging
NACT: Neoadjuvant chemotherapy
pCR: Pathological complete response
PET/CT: Positron emission tomography/computed tomography
ROC: Receiver operating characteristics
US: Ultrasound

## Declarations

### Guarantor

The scientific guarantor of this publication is associated professor Daniel Förnvik, Lund University.

### Statistics and biometry

No complex statistical methods were necessary for this paper.

### Study subjects or cohorts overlap

Some study subjects or cohorts have been previously reported in:

Published:

- Skarping I, Fornvik D, Heide-Jorgensen U, et al. Mammographic density changes during neoadjuvant breast cancer treatment: NeoDense, a prospective study in Sweden. Breast. 2020 Oct;53:33–41. (Overlap: This study is based on the prospective cohort included in the current study. In this study, we investigated mammographic density (using VolparaTM) and its association with pCR).

- Skarping I, Fornvik D, Sartor H, et al. Mammographic density is a potential predictive marker of pathological response after neoadjuvant chemotherapy in breast cancer. BMC Cancer. 2019 Dec 30;19(1):1272. (Overlap: This study is based on the retrospective cohort included in the current study. In this study, we investigated mammogrpohic density (using Volpara ^TM^) and its association with pCR)..

- Skarping I, Fornvik D, Heide-Jorgensen U, et al. Mammographic density as an image-based biomarker of therapy response in neoadjuvant-treated breast cancer patients. Cancer Causes Control. 2021 Mar;32(3):251–260. (Overlap: This study is based on the retrospective and prospective cohort included in the current study. In this study, we investigated mammographic density (BI-RADS) and its association with pCR).

- Skarping I, Fornvik D, Heide-Jorgensen U, et al. Neoadjuvant breast cancer treatment response; tumour size evaluation through different conventional imaging modalities in the NeoDense study. Acta Oncol. 2020 Dec;59(12):1528–1537. (Overlap: This study is based on the prospective cohort included in the current study. In this study, we investigated different aspects of conventional imaging (mammography, ultrasound and tomosynthesis) during neoadjuvant chemotherapy for breast cancer).

Submitted: Skarping I, Blaabjerg Pedersen S, Förnvik D, Zackrisson S, Borgquist S. The association between body mass index and pathological complete response in neoadjuvant-treated breast cancer patients. (Overlap: This study is based on the retrospective and prospective cohort included in the current study. In this study, we investigated body mass index and its association with pCR)

### Methodology

• prospective

• diagnostic or prognostic study, observational cohort study

• performed at one institution
